# Diagnostic performance of kinetic parameters of ultrafast breast MRI and their associations with immunohistopathological findings of breast carcinoma

**DOI:** 10.1038/s41598-026-55998-5

**Published:** 2026-06-02

**Authors:** Aya Kobayashi, Megumi Matsuda, Kanako Okada, Takuya Matsuda, Rui Ebihara, Hitomi Ochi, Naoto Kawaguchi, Yasuhiro Shiraishi, Yoshihiro Takimoto, Yasutaka Ihara, Yoshiaki Kamei, Mie Kurata, Riko Kitazawa, Teruhito Kido

**Affiliations:** 1 Department of Radiology, Ehime University Graduate School of Medicine, Toon, Ehime Japan; 2 Department of Radiology, Ehime Prefectural Central Hospital, Matsuyama, Ehime Japan; 3 Department of Medical Informatics, Ehime University Graduate School of Medicine, Toon, Ehime Japan; 4 Department of Radiology, Ehime University Hospital, Toon, Ehime Japan; 5 Clinical Research Promotion Unit, Clinical Therapeutic Trial Center, Ehime University Hospital, Toon, Ehime Japan; 6 Breast Center, Ehime University Hospital, Toon, Ehime Japan; 7 Department of Pathology, Ehime University Proteo-Science Center, Toon, Ehime Japan; 8 Department of Analytical Pathology, Ehime University Graduate School of Medicine, Toon, Ehime Japan; 9 Division of Diagnostic Pathology, Ehime University Hospital, Toon, Ehime Japan

**Keywords:** Breast, Ultrafast breast MRI, Kinetics, Pathological findings, Biomarkers, Cancer, Oncology

## Abstract

This study aimed to assess diagnostic values of kinetic parameters in ultrafast dynamic contrast magnetic resonance imaging. The diagnostic performance of ultrafast kinetic parameters (time to enhancement [TTE], maximum slope [MS], time to peak, peak enhancement [PE], and wash-in slope [WIS]), and their correlations with the immunohistopathological findings of breast cancer were evaluated for 101 histologically proven breast lesions. We found that the areas under the curves of five ultrafast kinetic parameters in masses (0.69–0.81) and for MS, PE, and WIS in non-mass enhancement (NME) (0.91–0.94) were comparable to those of Breast Imaging Reporting and Data System (BI-RADS) categorizations (mass: 0.85, NME: 0.83) without statistically significant differences. Values of ultrafast kinetic parameters differed significantly according to invasiveness (invasive vs. non-invasive: TTE, *p* = 0.029; MS, *p* = 0.040; PE, *p* = 0.032; and WIS, *p* = 0.009), immunohistopathological findings (high vs. low Ki-67: TTE, *p* = 0.020; MS, *p* = 0.004; PE, *p* = 0.003; and WIS, *p* < 0.001), hormone receptor (negative vs. positive: MS, *p* = 0.033; and WIS, *p* = 0.042), and triple-negative status (triple-negative vs. non-triple-negative: MS, *p* = 0.028; and WIS, *p* = 0.011) in breast cancer. In conclusion, ultrafast kinetic parameters demonstrate diagnostic performance comparable to that of BI-RADS categories for both mass and NME and may be associated with the immunohistopathological findings of breast cancer.

**Supplementary Information:**

The online version contains supplementary material available at 10.1038/s41598-026-55998-5.

## Introduction

Breast magnetic resonance imaging (MRI) has been widely used to differentiate between benign and malignant breast lesions^1^. The American College of Radiology’s Breast Imaging Reporting and Data System (BI-RADS) MRI lexicon, which relies on the morphological and kinetic analyses of conventional dynamic contrast-enhanced MRI (cDCE-MRI) data, is the most widely used guideline for describing and categorizing enhancing breast lesions^2^.

Kinetic analysis requires early (within the first 2 min) and delayed phase data^2^. Consequently, cDCE-MRI is usually performed for 4–5 min or longer, which may be time-consuming. The use of ultrafast dynamic contrast-enhanced MRI (UF-MRI) for breast imaging in clinical practice and research has increased recently^3,4^. Several studies have reported using reconstruction techniques, including view-sharing and compressed sensing (CS), to achieve high spatial and temporal resolution for UF-MRI^5,6^. The UF-MRI enables rapid image acquisition at multiple time points during the early phase of contrast enhancement (0–90 s after contrast agent injection) and facilitates the assessment of early inflow of the contrast agent in breast lesions^7^.

Several kinetic parameters—including maximum slope (MS), time to enhancement (TTE), time to peak (TTP), peak enhancement (PE), and wash-in slope (WIS)—have been proposed to facilitate the objective evaluation of kinetic information obtained from UF-MRI^8–10^. Among these, MS and TTE are the two most frequently used for evaluating the diagnostic performance of UF-MRI^11^. Recent studies have reported that the diagnostic performance of UF-MRI based on these kinetic parameters is comparable or superior to that of cDCE-MRI according to BI-RADS categorization^12–14^; however, the evidence is limited. Some previous studies have also reported associations between these kinetic parameters—particularly MS and TTE—and carcinoma invasiveness and immunohistopathological findings of breast cancers, including the level of Ki-67^13,14^ and receptor status related to intrinsic subtypes^7,15^. However, these associations have not been fully clarified. Therefore, this study aimed to evaluate the utility of UF-MRI kinetic parameters in differentiating benign from malignant breast lesions for each mass and non-mass enhancement (NME), and to determine the correlation of these parameters with the immunohistopathological findings of breast cancers.

## Methods

### Patient selection

Our institutional review board (ETHICS COMMITTEE of EHIME UNIVERSITY HOSPITAL) approved this retrospective study (registration number: 2307009). The requirement for written informed consent was waived because of the retrospective study design. Our study population comprised 163 patients who underwent breast MRI, including UF-MRI, between November 2022 and June 2024.

Overall, 64 patients were excluded for the following reasons: absence of an enhancing lesion on breast MRI (*n* = 32), previous neoadjuvant chemotherapy (*n* = 13), no histological diagnosis and clinical or radiological follow-up (*n* = 15), breast cystic lesion with insufficient solid part for setting the region of interest (ROI) to calculate UF-MRI parameters (*n* = 3), and loss of part of the cDCE-MRI data due to injector trouble (*n* = 1). Consequently, the study included 99 patients (mean age, 54 years; age range, 15–92 years) with 101 lesions (72 masses and 29 NMEs). The histopathological diagnoses of all 101 breast lesions were confirmed based on the specimens obtained during surgery or biopsy. Of the 101 breast lesions, 57, 36, 1, and 7 were confirmed based on the findings of surgery, core needle biopsy, mammotome biopsy, and vacuum-assisted biopsy, respectively. MRI was performed before or at least 2 weeks after the biopsy to prevent artifacts^16^.

### MRI acquisition

**Fig. 1:**
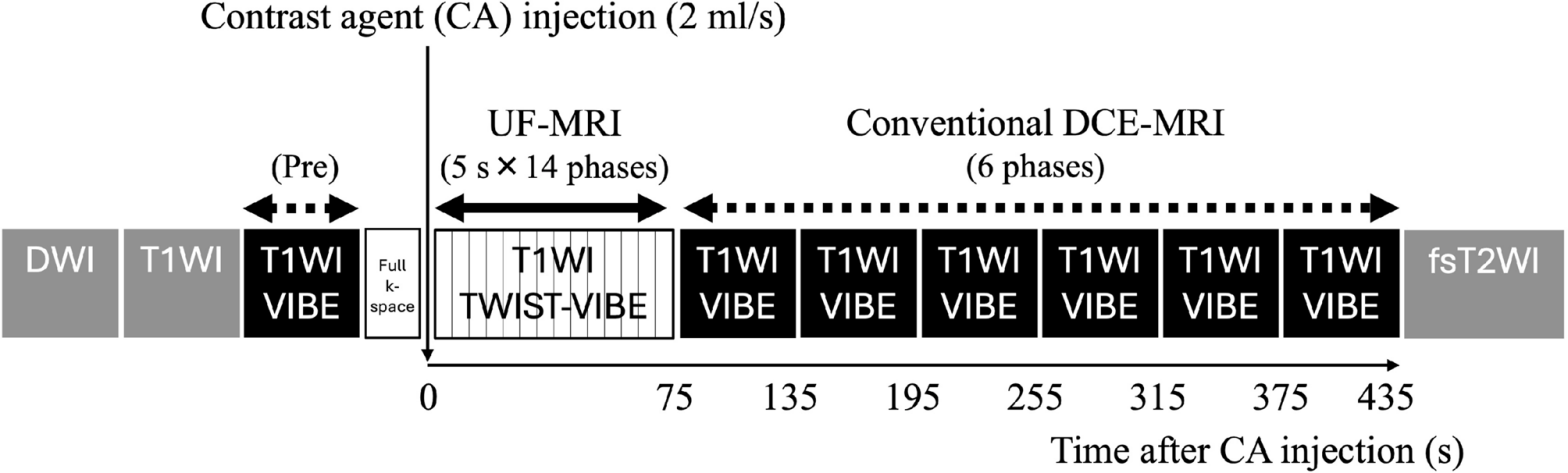
Breast MRI protocol. Axial diffusion-weighted images based on single-shot EPI, axial three-dimensional gradient-echo T1-weighted images, and one pre-contrast-enhanced three-dimensional fat-suppressed axial T1-weighted image using a VIBE sequence for cDCE study were obtained before the UF-MRI sequence. The coronal and axial fat-suppressed T2-weighted sequences were obtained after the cDCE study. cDCE: conventional dynamic contrast-enhanced; EPI: echo-planar imaging; MRI: magnetic resonance imaging; UF: ultrafast; VIBE: volume interpolated breath-hold examination.

All examinations were performed using a 3-T MR system (MAGNETOM Skyra, Siemens Healthcare Erlangen, Germany) and an 18-channel phased array dedicated breast coil. The contrast agent (gadoterate meglumine, 0.2 mmol/kg; Magnescope, Guerbet, Tokyo, Japan) was administered intravenously. This was followed by a 30-mL saline flush at a rate of 2.0 mL/s. UF-MRI was performed using time-resolved angiography with the interleaved stochastic trajectories (TWIST) volume interpolated breath-hold examination (VIBE) technique, which improves temporal resolution while preserving its spatial resolution. Before the UF-MRI sequence, axial diffusion-weighted images based on single-shot echo-planar imaging, axial gradient-echo T1-weighted image, and one pre-contrast-enhanced three-dimensional fat-suppressed axial T1-weighted image using the VIBE sequence for cDCE study were obtained. The first of the 15 UF-MRI series was obtained before the contrast agent injection. The remaining 14 series, which had a temporal resolution of 5.0 s, were obtained immediately after and during the contrast agent injection. The following imaging parameters of UF-MRI were applied: repetition time (TR)/echo time (TE), 5.9/2.87 ms; flip angle (FA), 10°; field of view (FOV), 350 × 350 mm; number of slice, 72; slice thickness, 2.0 mm; reconstruction voxel size, 0.9 × 0.9 × 2.0 mm; acquisition matrix, 384 × 250; Controlled Aliasing in Parallel Imaging Results in Higher Acceleration (CAIPIRINHA) acceleration factor, 4; and fat suppression via water excitation. The TWIST k-space view-sharing parameters for regions A and B were 20% and 25%, respectively, except for the first pre-contrast phase (12.7 s, full k-space sampling). A and B represent the percentages of the central and peripheral k-space regions, respectively. The first post-contrast sequence using a three-dimensional fat-suppressed VIBE sequence for cDCE-MRI was acquired 90 s after injecting the contrast agent. Subsequently, the five series were obtained at 60-s intervals for the cDCE study. The k-space centers for the six contrast phases were acquired at 115.9, 175.9, 235.9, 295.9, 355.9, and 415.9 s after contrast agent injection, respectively. The cDCE-MRI parameters were as follows: TR/TE, 3.3/1.26 ms; FA, 15°; FOV, 350 × 350 mm; number of slice, 208; slice thickness, 0.7 mm; reconstruction voxel size, 0.9 × 0.9 × 0.7 mm; acquisition matrix, 384 × 269; generalized autocalibrating partial parallel acquisition (GRAPPA) acceleration factor, 3; and fat suppression using Spectral Attenuated Inversion Recovery (SPAIR). The orientations of UF-MRI and cDCE-MRI were axial images. Coronal sequences for evaluating axillary lesions and axial fat-suppressed T2-weighted images were obtained after the cDCE study. Our breast MRI protocol is provided in Fig. 1.

### Interpretation of cDCE-MRI

The breast lesions were morphologically evaluated using SYNAPSE SAI viewer (Version 2.4, FUJIFILM Corporation, Tokyo, Japan; https://www.fujifilm.com/jp/ja/healthcare/healthcare-it/it-imaging/sai-viewer), a medical viewing system. Two radiologists (observers 1 and 2 with 17 and 10 years of breast MRI experience, respectively) independently classified the enhanced lesions identified on cDCE-MRI as masses (*n* = 72) or NMEs (*n* = 29). They proceeded to categorize these lesions using the MRI lexicon of BI-RADS (5th edition). The radiologists were provided with the mammography and ultrasonography (US) findings; however, they were blinded to the final pathology. For deciding BI-RADS category, the radiologists retrospectively reviewed and evaluated morphologic features including shape and margin, internal enhancement characteristics, and time–signal intensity curve (TIC) patterns of cDCE-MRI in the initial and delayed phases for the masses and the internal enhancement patterns and distribution for NMEs. The TIC was obtained by drawing a circular ROI of > 3 pixels on the most suspicious region of enhancement within the mass lesion, which were usually located at the tumor margin. The ROI was set to avoid necrosis, cystic areas, and blood vessels. A specific analysis software Volume Analyzer SYNAPSE VINCENT (Version 6.8.0001, FUJIFILM Corporation, Tokyo, Japan; https://www.fujifilm.com/jp/ja/healthcare/healthcare-it/it-3d/vincent) was used to determine the TIC patterns of the breast mass lesions^17^. The two observers independently placed ROIs to determine the TIC patterns. All enrolled masses and NME lesions were categorized using BI-RADS scores (3–5). The differences in BI-RADS classifications between the two observers were resolved through consensus. Breast lesions categorized as 4 and 5 were classified as malignant, and those categorized as 3 were considered benign. Observer 1 and 2 measured the lesion size independently, with the mean of their measured values being considered the lesion size. The lesion size was determined using its largest diameter in three reformatted planes (i.e., sagittal, axial, and coronal) on cDCE-MRI.

### Interpretation of UF-MRI

UF-MRI data were analyzed using the SYNAPSE VINCENT software. Observer 1, and observer 3 (who has 5 years of breast MRI experience) independently determined the start time of aortic enhancement by visually assessing the aorta at the level of the main trunk of the pulmonary artery. The SI of the breast lesion was automatically converted to a percentage change for relative enhancement based on the pre-contrast SI from the TIC for UF-MRI. Observer 1 and observer 3 independently selected a circular ROI of > 3 pixels for each breast-enhancing lesion including mass and NME on the UF-MRI to evaluate the strongest and fastest enhancement. The ROIs were placed to be completely within either mass or NME. Additionally, observers 1 and 3 determined TTE, MS, TTP, PE, and WIS based on the TIC for the UF-MRI of the lesions and the start time of aortic enhancement^3,9,10^. TTE [s] was defined as the time interval between the onset of aortic enhancement and the start of enhancement of the breast lesion. MS [%/s] was calculated based on the maximum change in the relative enhancement of the breast lesion between two time points. TTP [s] was defined as the time interval from the onset of breast lesion enhancement to the point of maximal relative enhancement on UF-MRI. PE [%] was defined as the maximal relative enhancement of the breast lesion on UF-MRI. WIS [%/s] was calculated as PE divided by TTP.

### Pathological assessment

The histopathological findings from biopsies or surgical specimens were extracted from the pathology reports prepared at the time of treatment. Two pathologists independently made the diagnoses, one of whom (with 32 years of experience in breast lesion diagnosis) confirmed the final diagnosis. Hematoxylin and eosin staining was performed for the pathological diagnosis. The expression levels of estrogen receptor (ER), progesterone receptor (PgR), human epidermal growth factor receptor 2 (HER2), and Ki-67 in each patient with invasive breast carcinoma were determined using streptavidin-peroxidase immunohistochemistry (IHC). ER and PgR positivity were defined as the presence of 1% positively stained nuclei in 10 high-power fields^18^. ER and/or PgR positivity was considered hormone receptor (HR)-positive, while both ER and PgR negativity were considered HR-negative. HER2 positivity was defined as an IHC HER2 score of 3 or as gene amplification observed by fluorescence in situ hybridization in tumors with an IHC HER2 score of 2. Triple-negative breast cancer (TNBC) was classified as HR-negative and HER2-negative. Ki-67 expression was classified into the following two groups: a low Ki-67 group defined as < 14% Ki-67 detection, and a high Ki-67 group defined as ≥ 14% Ki-67 detection.

### Statistical analysis

Continuous data are summarized as medians and interquartile ranges. The lesion sizes are described mean and range. The Mann–Whitney U test was used to compare the UF-MRI kinetic parameters between benign and malignant breast lesions, as well as invasiveness and immunohistopathological findings of breast cancer. Regarding the differentiation of benign and malignant lesions for mass and NME using UF-MRI kinetic parameters, we further analyzed details such as the determination of cut-off values and evaluation of diagnostic performance only for the UF-MRI kinetic parameters that showed significant differences between benign and malignant lesions in the Mann–Whitney U test. Their effectiveness for differentiating between benign and malignant lesions with mass and NME was determined using receiver operating characteristic (ROC) curve analysis. The area under the curve (AUC) was reported as the mean and 95% confidence interval (CI).

Diagnostic performances of BI-RADS and UF-MRI kinetic parameters were assessed using sensitivity, specificity, positive predictive value (PPV), negative predictive value (NPV), and accuracy. For the BI-RADS diagnosis, categories 4 and 5 were defined as positive for malignancy; for the UF-MRI kinetic parameters, cut-off values for distinguishing between malignant and benign lesions were determined based on the highest sensitivity and specificity (maximal Youden index, defined as sensitivity plus specificity minus 1) from the ROC curves.

To compare sensitivity, specificity, and accuracy of the six indicators (BI-RADS and TTE, MS, TTP, PE, and WIS) and AUCs of BI-RADS and UF-MRI kinetic parameters for mass and NME, we first applied Cochran’s Q test as a global test of equality of paired proportions. When a global test was statistically significant (*p* < 0.05), we conducted pre-specified comparisons of sensitivity, specificity, and accuracy between BI-RADS and each UF-MRI kinetic parameter using McNemar’s test and AUCs of BI-RADS and UF-MRI kinetic parameters for mass and NME using DeLong’s test^19^.

Because two patients had two lesions each, we re-analyzed the diagnostic performance of BI-RADS and UF-MRI kinetic parameters for masses and NMEs and enrolled only the larger lesions in these two patients (i.e., two lesions in total were removed, and 99 lesions in 99 patients were included in the re-analysis) in the evaluation of the potential impact of within-patient correlation on the results.

Interobserver agreements for BI-RADS category classification and descriptors for masses and NMEs were determined using the weighted kappa statistic (> 0.81, excellent agreement; 0.61–0.80, good agreement; 0.41–0.60, moderate agreement; 0.21–0.40, fair agreement; and ≤ 0.20, poor agreement). The interobserver agreement for the five UF-MRI kinetic parameters was determined using the intraclass correlation coefficient (ICC) (1.0, perfect agreement; 0.81–0.99, almost perfect agreement; 0.61–0.80, substantial agreement; 0.41–0.60, moderate agreement; 0.21–0.40, fair agreement; and ≤ 0.20, slight agreement)^20^.

The global, McNemar’s, and DeLong’s tests were performed using the R software, version 4.4.2 (R Core Team)^21^. Other statistical analyses were performed using IBM SPSS Statistics for Windows, version 28.0 (IBM Corp., Armonk, N.Y., USA). Statistical significance was set at *p* < 0.05.

## Results

Among the 101 breast lesions in 99 patients, 72 lesions in 71 patients were masses (benign; 18, malignant; 54), while 29 lesions in 29 patients were NMEs (benign; 5, malignant; 24). Two of the 99 patients had two breast lesions: 1 had a lesion in each breast (a mass and NME in the right and left breasts, respectively), and 1 had two mass lesions in the left breast. For masses, the mean sizes on cDCE-MRI of malignant and benign lesions were 18.0 mm (range: 6.9–59.2 mm) and 14.8 mm (range: 6.4–41.3 mm), respectively. There was no significant difference between malignant and benign lesions (*p* = 0.222). For NMEs, the mean sizes of malignant and benign lesions were 29.2 mm (range: 4.8–93.9 mm) and 12.5 mm (range: 5.6–17.6 mm), respectively. Malignant lesions were significantly larger than benign lesions (*p* = 0.032).

### Differences in the UF-MRI kinetic parameters for mass and NME between malignant and benign lesions

The interobserver agreements for the UF-MRI kinetic parameters were evaluated. The kinetic parameters for the 101 lesions showed substantial or almost perfect interobserver agreement. The ICCs for TTE, MS, TTP, PE, and WIS were 0.864 (95% CI: 0.81–0.91), 0.922 (95% CI: 0.89–0.95), 0.778 (95% CI: 0.69–0.84), 0.936 (95% CI: 0.91–0.96), and 0.921 (95% CI: 0.89–0.95), respectively. The kinetic parameters determined by observer 1 were used for the subsequent evaluations. Table 1 summarizes the differences in the UF-MRI kinetic parameters for both masses and NMEs between benign and malignant lesions. For mass lesions, the MS (17.0%/s vs. 8.5%/s), PE (221.8% vs. 136.9%), and WIS (5.7%/s vs. 2.8%/s) were significantly larger (all *p* < 0.001), while the TTE (*p* = 0.011, 5.0 s vs. 10.1 s) and TTP (*p* < 0.001, 40.3 s vs. 50.3 s) were significantly smaller for malignant lesions than those for benign lesions. For NMEs, the MS (*p* = 0.001, 11.7%/s vs. 4.2%/s), PE (*p* = 0.003, 167.6% vs. 66.6%), and WIS (*p* < 0.001, 3.8%/s vs. 1.3%/s) were significantly larger for malignant lesions than those for benign lesions. In contrast, no significant difference was observed in TTE (*p* = 0.145, 5.0 s vs. 10.0 s) and TTP (*p* = 0.889, 45.3 s vs. 45.3 s) between benign and malignant lesions for NMEs.

**Table 1:** Differences in UF-MRI kinetic parameters for masses and NMEs between benign and malignant lesions.

Parameters in UF-MRI	Malignant	Benign	*P*-value	AUC (95% CI)	Cut-off value
**Mass**					
TTE (s)	5.0 (5.0, 10.0)	10.1 (5.0, 15.0)	*0.011*	0.69 (0.50–0.84)	10.05
MS (%/s)	17.0 (12.2, 22.0)	8.5 (7.0, 11.0)	*< 0.001*	0.81 (0.67–0.93)	10.20
TTP (s)	40.3 (34.2, 45.3)	50.3 (45.3, 50.3)	*< 0.001*	0.77 (0.63–0.89)	47.30
PE (%)	221.8 (170.0, 273.5)	136.9 (97.3, 191.1)	*< 0.001*	0.76 (0.61–0.90)	145.15
WIS (%/s)	5.7 (4.2, 7.9)	2.8 (2.2, 4.2)	*< 0.001*	0.79 (0.64–0.91)	3.03
AUC (95% CI) of BI-RADS for masses				0.85 (0.78–0.91)	
**NME**					
TTE (s)	5.0 (5.0, 10.1)	10.0 (9.1, 15.1)	0.145		
MS (%/s)	11.7 (7.9, 21.8)	4.2 (3.9, 4.4)	*0.001*	0.93 (0.83–1.00)	6.73
TTP (s)	45.3 (40.3, 50.3)	45.3 (45.3, 50.3)	0.889		
PE (%)	167.6 (114.8, 259.1)	66.6 (57.9, 68.5)	*0.003*	0.91 (0.75–1.00)	77.85
WIS (%/s)	3.8 (2.5, 6.0)	1.3 (1.3, 1.5)	*< 0.001*	0.94 (0.83–1.00)	1.54
AUC (95% CI) of BI-RADS for NME				0.83 (0.65–0.98)	

Data are expressed as the median (interquartile range). P-values < 0.05 were considered statistically significant and are underlined. AUC: area under the curve; CI: confidence interval; BI-RADS: Breast Imaging Reporting and Data System; MS: maximum slope; NME: non-mass enhancement; PE: peak enhancement; TTE: time to enhancement; TTP: time to peak; UF-MRI: ultrafast dynamic contrast-enhanced MRI; WIS: wash-in slope.

### Diagnostic performance of BI-RADS and UF-MRI kinetic parameters for masses and NMEs

**Fig. 2:**
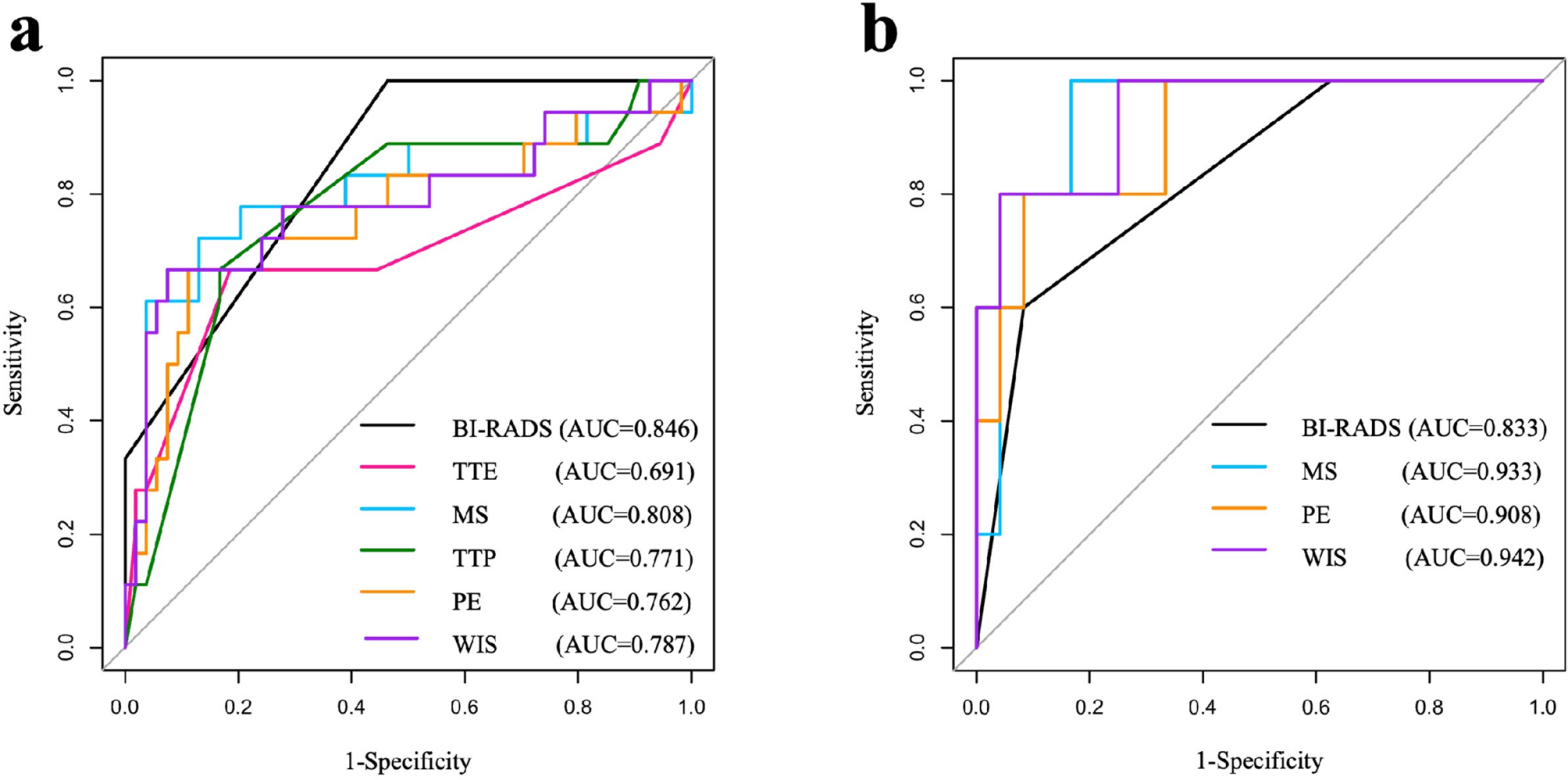
ROC curves and AUCs for BI-RADS and cut-off values of the UF-MRI kinetic parameters for masses (**a**) and NMEs (**b**). AUC: area under the curve; BI-RADS: Breast Imaging Reporting and Data System; MRI: magnetic resonance imaging; NME: non-mass enhancement; ROC: receiver operating characteristics; UF: ultrafast.

Table 2 presents the diagnostic performance of BI-RADS and the cut-off values of the UF-MRI kinetic parameters for both masses and NMEs. Figure 2 shows the ROC curves for the cut-off values of UF-MRI kinetic parameters and BI-RADS for the mass and NME. For mass lesions, ROC analysis revealed an AUC of 0.85 for BI-RADS (Table 1; Fig. 2a). Twenty-five (67.6%) of the 37 category 4 lesions were malignant, and 12 (32.4%) were benign (8 fibroadenomas, 2 intraductal papillomas, 1 tubular adenoma, and 1 benign epithelial lesion). The PPV and accuracy were both 67.6% (95% CI: 52.48–82.65). All six category 3 lesions were diagnosed as benign, while all 29 category 5 lesions were diagnosed as malignant. For NMEs, ROC analysis revealed an AUC of 0.83 for BI-RADS (Table 1; Fig. 2b). Of the 15 category 4 lesions, 13 (86.7%) were diagnosed as malignant, while 2 (13.3%) were diagnosed as benign (1 fibroadenoma and 1 benign epithelial lesion). The PPV and accuracy were both 86.7% (95% CI: 69.46–103.87). Of the 5 category 3 lesions, 2 (40.0%) were diagnosed as malignant (1 ductal carcinoma in situ [DCIS] and 1 invasive ductal carcinoma [IDC]), and 3 (60.0%) were benign. The NPV and accuracy were both 60.0% (95% CI: 17.06–102.94). All nine category 5 lesions were diagnosed as malignant. All enrolled breast masses were classified as malignant or benign based on the cut-off values of the UF-MRI kinetic parameters that showed significant differences in the Mann–Whitney U test. The optimal cut-off values of TTE, MS, TTP, PE, and WIS for differentiating between malignant and benign breast masses were 10.05 s, 10.20%/s, 47.30 s, 145.15%, and 3.03%/s, respectively (Table 1).

**Table 2:** Diagnostic performance comparison of cut-off values of UF-MRI kinetic parameters versus BI-RADS for mass and NME.

	TP	FN	FP	TN	Sensitivity (%) (Difference ref. BI-RADS)	*P*-value	Specificity (%) (Difference ref. BI-RADS)	*P*-value	Accuracy (%) (Difference ref. BI-RADS)	*P*-value
Mass										
Global test						*0.012*		*0.022*		0.619
McNemar’s test										
BI-RADS	54	0	12	6	100		33.3		83.3	
TTE	44	10	6	12	81.5 (-18.5)	*0.002*	66.7 (33.4)	*0.014*	77.8 (-5.5)	
MS	47	7	5	13	87.0 (-13.0)	*0.008*	72.2 (38.9)	*0.008*	83.3 (0.0)	
TTP	45	9	6	12	83.3 (-16.7)	*0.003*	66.7 (33.4)	*0.034*	79.2 (-4.1)	
PE	48	6	6	12	88.9 (-11.1)	*0.014*	66.7 (33.4)	*0.014*	83.3 (0.0)	
WIS	50	4	6	12	92.6 (-7.4)	*0.046*	66.7 (33.4)	*0.014*	86.1 (2.8)	
NME										
Global test						0.159		0.494		0.666
McNemar’s test										
BI-RADS	22	2	2	3	91.7		60		86.2	
MS	20	4	0	5	83.3 (-8.4)		100 (40)		86.2 (0.0)	
PE	22	2	1	4	91.7 (0.0)		80 (20)		89.7 (3.5)	
WIS	23	1	1	4	95.8 (4.1)		80 (20)		93.1 (6.9)	

Cut-off values for Mass: TTE, 10.05 s; MS, 10.20%/s; TTP, 47.30 s; PE, 145.15%; WIS, 3.03%/s.

Cut-off values for NME: MS, 6.73%/s; PE, 77.85%; WIS, 1.54%/s. P-values < 0.05 were considered statistically significant and are underlined. BI-RADS: Breast Imaging Reporting and Data System; FN: False Negative; FP: False Positive; MS: maximum slope; NME: non-mass enhancement; NPV: Negative Predictive Value; PE: peak enhancement; PPV: Positive Predictive Value; TN: True Negative; TP: True Positive; TTE: time to enhancement; TTP: time to peak; UF-MRI: ultrafast dynamic contrast-enhanced MRI; WIS: wash-in slope.

For mass lesions, the global test indicated significant differences in sensitivity among the six indicators (global test, *p* = 0.012). In the pre-specified McNemar comparisons, the sensitivities of all UF-MRI kinetic parameters at their optimal cut-off values were significantly lower than those of BI-RADS (TTE vs. BI-RADS: 81.5%, *p* = 0.002; MS vs. BI-RADS: 87.0%, *p* = 0.008; TTP vs. BI-RADS: 83.3%, *p* = 0.003; PE vs. BI-RADS: 88.9%, *p* = 0.014; WIS vs. BI-RADS: 92.6%, *p* = 0.046). Conversely, specificities differed significantly in the global test (*p* = 0.022) and were higher for UF-MRI kinetic parameters than for BI-RADS (TTE vs. BI-RADS: 66.7%, *p* = 0.014; MS vs. BI-RADS: 72.2%, *p* = 0.008; TTP vs. BI-RADS: 66.7%, *p* = 0.034; PE vs. BI-RADS: 66.7%, *p* = 0.014; WIS vs. BI-RADS: 66.7%, *p* = 0.014). In contrast, accuracies did not differ significantly among the six indicators (global test, *p* = 0.619). For mass lesions, the UF-MRI kinetic parameters yielded fewer true positives (BI-RADS: 54; TTE: 44; MS: 47; TTP: 45; PE: 48; and WIS: 50) and false positives (BI-RADS: 12; TTE: 6; MS: 5; TTP: 6; PE: 6; and WIS: 6) and more false negatives (BI-RADS: 0; TTE: 10; MS: 7; TTP: 9; PE: 6; and WIS: 4) and true negatives (BI-RADS: 6; TTE: 12; MS: 13; TTP: 12; PE: 12; and WIS: 12) than BI-RADS (Table 2).

Table 3 presents the number of true positives and negatives according to the BI-RADS and cut-off values of UF-MRI kinetic parameters for each histological type. Of the 54 malignant masses, 3 were DCIS. The number of true positives for these three DCIS was 3, 1, 0, 3, 2, and 3 in BI-RADS, TTE, MS, TTP, PE, and WIS, respectively, and tended to be more for TTP, PE, and WIS than for MS and TTE among the UF-MRI kinetic parameters. Of the 18 benign mass lesions, 10 were fibroadenoma. The number of true negatives for these fibroadenoma was 2, 6, 7, 9, 6, and 6 in BI-RADS, TTE, MS, TTP, PE, and WIS, respectively, and tended to be more for the UF-MRI kinetic parameters than for BI-RADS. In one tubular adenoma, the number of true positives was zero for both BI-RADS and UF-MRI kinetic parameters. The ROC analysis revealed that the TTE, MS, TTP, PE, and WIS had AUCs ranging from 0.69 to 0.81 for masses (Table 1; Fig. 2a). A global comparison of AUCs across six indicators was statistically significant (*p* < 0.001). However, the DeLong’s test showed that AUCs of UF-MRI kinetic parameters were comparable to that of BI-RADS (0.85) for detecting masses (TTE vs. BI-RADS: *p* = 0.053; MS vs. BI-RADS: *p* = 0.579; TTP vs. BI-RADS: *p* = 0.297; PE vs. BI-RADS: *p* = 0.232; and WIS vs. BI-RADS: *p* = 0.399).

**Table 3:** Number of prediction outcomes according to the BI-RADS and cut-off values of UF-MRI kinetic parameters for each histological type.

**Mass**						
**Malignant**	Number of true positives/false negatives					
Total 54 lesions	BI-RADS	TTE	MS	TTP	PE	WIS
IDC (*n* = 41)	41/0	34/7	38/3	33/8	36/5	37/4
Apocrine carcinoma (*n* = 3)	3/0	3/0	3/0	3/0	3/0	3/0
Mucinous carcinoma (*n* = 3)	3/0	3/0	2/1	3/0	3/0	3/0
DCIS (*n* = 3)	3/0	1/2	0/3	3/0	2/1	3/0
Medullary carcinoma (*n* = 2)	2/0	1/1	2/0	1/1	2/0	2/0
LCIS (*n* = 1)	1/0	1/0	1/0	1/0	1/0	1/0
ILC (*n* = 1)	1/0	1/0	1/0	1/0	1/0	1/0
**Benign**	Number of true negatives/false positives					
Total 18 lesions						
Fibroadenoma (*n* = 10)	2/8	6/4	7/3	9/1	6/4	6/4
Intraductal papilloma (*n* = 3)	1/2	2/1	2/1	1/2	2/1	2/1
Fibrocystic disease (*n* = 2)	2/0	2/0	2/0	1/1	2/0	2/0
Hamartoma (*n* = 1)	1/0	1/0	1/0	1/0	1/0	1/0
Benign epithelial lesion (*n* = 1)	0/1	1/0	1/0	0/1	1/0	1/0
Tubular adenoma (*n* = 1)	0/1	0/1	0/1	0/1	0/1	0/1
**NME**						
**Malignant**	Number of true positives/false negatives					
Total 24 lesions	BI-RADS	MS	PE	WIS		
DCIS (*n* = 12)	11/1	10/2	11/1	12/0		
IDC (*n* = 9)	8/1	8/1	8/1	8/1		
ILC (*n* = 2)	2/0	1/1	2/0	2/0		
Apocrine carcinoma (*n* = 1)	1/0	1/0	1/0	1/0		
**Benign**	Number of true negatives/false positives					
Total of 5 lesions						
Benign epithelial lesion (*n* = 3)	2/1	3/0	2/1	2/1		
Fibrocystic disease (*n* = 1)	1/0	1/0	1/0	1/0		
Fibro adenoma (*n* = 1)	0/1	1/0	1/0	1/0		

BI-RADS: Breast Imaging Reporting and Data System; MS: maximum slope; NME: non-mass enhancement; PE: peak enhancement; TTE: time to enhancement, TTP: time to peak; WIS: wash-in slope; IDC: invasive ductal carcinoma; DCIS: ductal carcinoma in situ; LCIS: lobular carcinoma in situ; ILC: invasive lobular carcinoma.

For NMEs, ROC analysis revealed an AUC of 0.83 for BI-RADS (Table 1; Fig. 2b). All enrolled breast NMEs were differentiated into malignant and benign based on the cut-off values of MS, PE, and WIS, which showed significant differences in the Mann–Whitney U test. The optimal cut-off values of MS, PE, and WIS for differentiating malignant from benign breast lesions were 6.73%/s, 77.85%, and 1.54%/s, respectively (Table 1). For NMEs, the global test showed no significant differences in sensitivity (*p* = 0.159), specificity (*p* = 0.494), or accuracy (*p* = 0.666) among the four indicators—including BI-RADS and UF-MRI kinetic parameters—using their optimal cut-off values (Table 2).

For NMEs, the numbers of true positive (BI-RADS: 22; MS: 20; PE: 22; and WIS: 23), false positive (BI-RADS: 2; MS: 0; PE: 1; and WIS: 1), false negative (BI-RADS: 2; MS: 4; PE: 2; and WIS: 1), and true negative (BI-RADS: 3; MS: 5; PE: 4; and WIS: 4) were similar to those of BI-RADS in MS, PE, and WIS (Table 2).

The ROC analysis revealed that the MS, PE, and WIS had AUCs ranging from 0.91 to 0.94, which tended to be slightly higher than that of BI-RADS (0.83) for NMEs (Table 1; Fig. 2b). The global comparison of AUCs across BI-RADS, MS, PE, and WIS was not statistically significant (*p* = 0.133).

The Cohen’s weighted kappa values for BI-RADS category classification for masses and NMEs were 0.84 (95% CI: 0.73–0.95) and 0.72 (95% CI: 0.51–0.94), respectively, indicating excellent and good agreement.

The Cohen’s weighted kappa values of BI-RADS descriptors for masses and NMEs were evaluated. For masses, the Cohen’s weighted kappa values were assessed for five BI-RADS descriptors—shape, margin, internal enhancement characteristics, initial and delayed-phase kinetic curve assessment—and were 0.72 (95% CI: 0.56–0.88), 0.76 (95% CI: 0.64–0.88), 0.71 (95% CI: 0.59–0.82), 0.73 (95% CI: 0.46–1.0), and 0.84 (95% CI: 0.73–0.95), respectively, indicating excellent and good agreement. For NMEs, the Cohen’s weighted kappa values were assessed for two BI-RADS descriptors—internal enhancement patterns and distribution—and were 0.72 (95% CI: 0.54–0.91) and 0.74 (95% CI: 0.53–0.95), respectively, indicating good agreement.

**Fig. 3:**
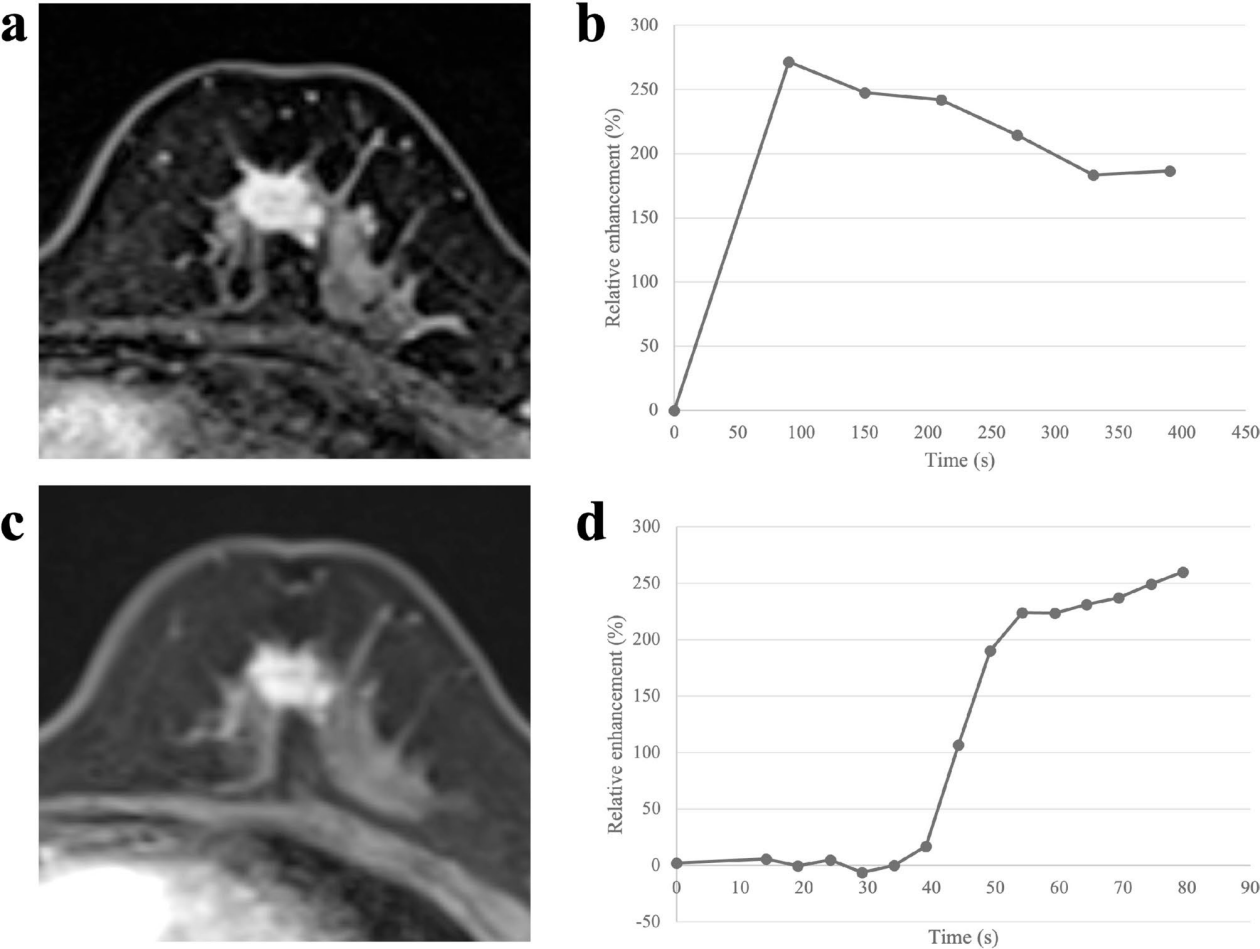
A representative case of a 55-year-old woman with IDC in the left breast. (**a**) Early phase cDCE-MRI. (**b**) TIC of cDCE-MRI. (**c**) UF-MRI. (**d**) TIC of UF-MRI. Axial early phase cDCE-MRI (**a**) shows the mass lesion with an irregular shape, spiculated margin, and heterogeneous internal enhancement. The TIC of the cDCE-MRI (**b**) shows a fast-washout pattern. This mass is classified as BI-RADS category 5. The TTE, MS, TTP, PE, and WIS of the mass lesion obtained from the TIC of UF-MRI (**d**) were 5.0 s, 17.59%/s, 45.3 s, 260%, and 5.74%/s, respectively. These values of UF-MRI parameters suggested malignant kinetic patterns based on the cut-off values for differentiating between benign and malignant breast mass lesions. BI-RADS: Breast Imaging Reporting and Data System; cDCE: conventional dynamic contrast-enhanced; IDC: invasive ductal carcinoma; MRI: magnetic resonance imaging; MS: maximum slope; PE: peak enhancement; TIC: time-signal intensity curve; TTE: time to enhancement; TTP: time to peak; UF: ultrafast; WIS: wash-in slope.

**Fig. 4:**
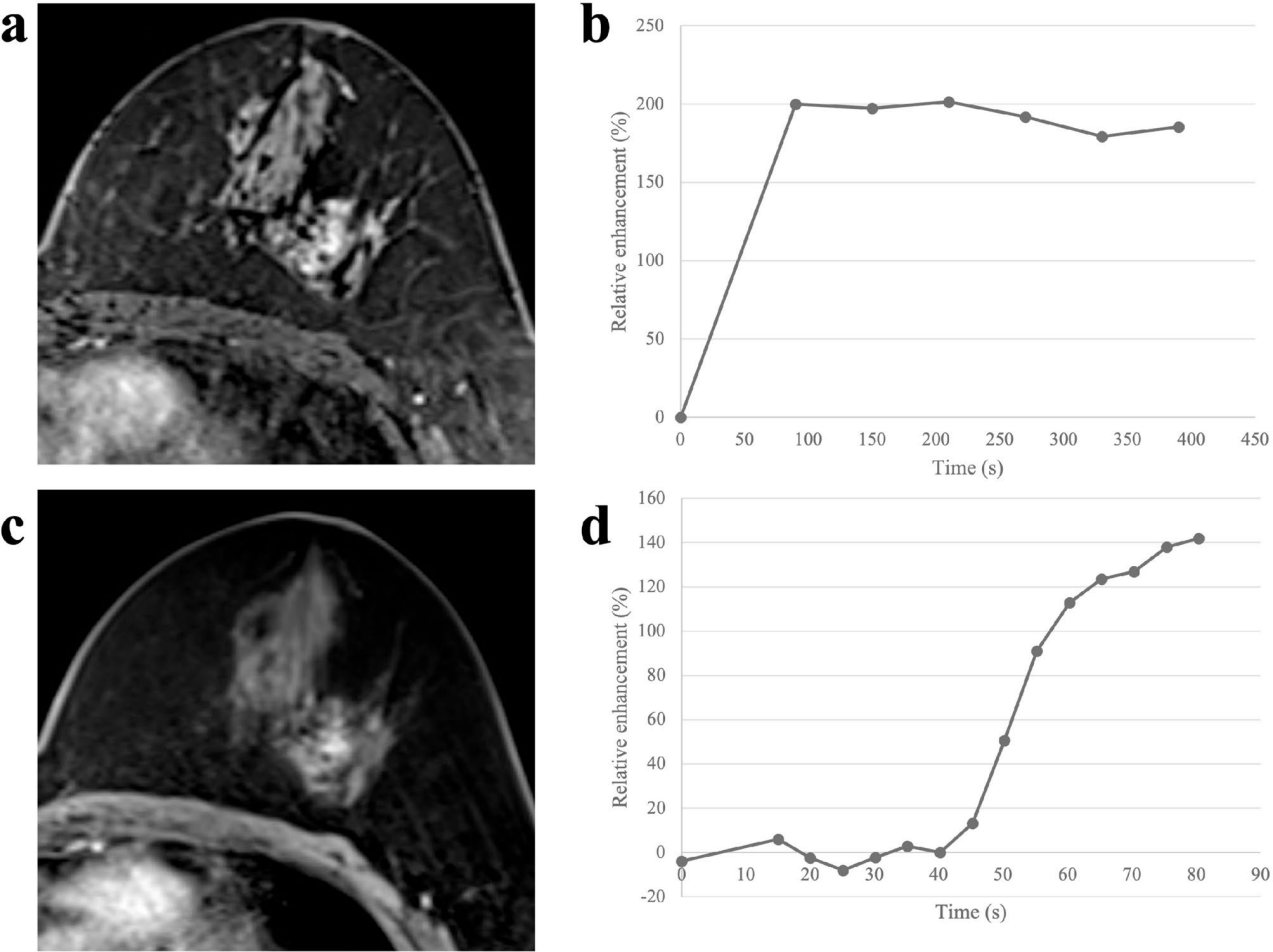
A representative case of a 56-year-old woman with DCIS in the left breast. (**a**) Early phase cDCE-MRI. (**b**) TIC of cDCE-MRI. (**c**) UF-MRI. (**d**) TIC of UF-MRI. The axial early phase cDCE-MRI (**a**) shows NME with a clustered ring pattern and segmental distribution. The TIC of the cDCE-MRI (**b**) shows a fast-plateau pattern. This NME is classified as BI-RADS category 5. The TTE, MS, TTP, PE, and WIS of the NME determined from the TIC of the UF-MRI (**d**) were 10.1 s, 8.08%/s, 45.3 s, 142%, and 3.13%/s, respectively. MS, PE, and WIS suggested malignant kinetic patterns based on the cut-off values for differentiating between benign and malignant breast lesions for NME. BI-RADS: Breast Imaging Reporting and Data System; cDCE: conventional dynamic contrast-enhanced; DCIS: ductal carcinoma in situ; MRI: magnetic resonance imaging; MS: maximum slope; NME: non-mass enhancement; PE: peak enhancement; TIC: time-signal intensity curve; TTE: time to enhancement; TTP: time to peak; UF: ultrafast; WIS: wash-in slope.

Representative cases of IDC and DCIS are provided to illustrate the cDCE-MRI findings and UF-MRI kinetic parameters (Figs. 3 and 4). For the diagnostic performance of BI-RADS and UF-MRI kinetic parameters for masses and NMEs, the difference in the original results analyzed for all lesions (101 lesions in 99 patients) and re-analysis results including only larger lesions in the two patients with multiple lesions (99 lesions in 99 patients) was negligible. The re-analysis results are shown in the supplementary materials.

### Correlation between kinetic parameters in UF-MRI and invasiveness and immunohistopathological findings of breast carcinoma

Of the enrolled 101 breast lesions, 78 were breast carcinoma, which included 62 invasive and 16 non-invasive carcinomas. Table 4 presents the differences in the UF-MRI kinetic parameters for invasiveness and each immunohistopathological finding of breast carcinoma. For invasive carcinoma, the TTE (*p* = 0.029, invasive vs. non-invasive: 5.0 s vs. 7.6 s) was significantly smaller, while the MS (*p* = 0.040, invasive vs. non-invasive: 17.0%/s vs. 9.2%/s), PE (*p* = 0.032, invasive vs. non-invasive: 221.8% vs. 154.9%), and WIS (*p* = 0.009, invasive vs. non-invasive: 5.7%/s vs. 3.4%/s) were significantly larger than those of non-invasive carcinoma. No significant difference was observed between invasive and non-invasive carcinomas for TTP (*p* = 0.201, invasive vs. non-invasive: 42.8 s vs. 45.3 s). For the 62 invasive breast carcinomas, correlation between immunohistopathological findings—including Ki-67 status (high; ≥14% vs. low; <14%), HR status (negative vs. positive), HER2 status (positive vs. negative), and TNBC (TNBC vs. non-TNBC) and UF-MRI kinetic parameters—were evaluated. For the high Ki-67 group, the TTE (*p* = 0.020, high vs. low: 5.0 s vs. 5.1 s) was significantly smaller, while the MS (*p* = 0.004, high vs. low: 19.1%/s vs. 12.4%/s), PE (*p* = 0.003, high vs. low: 241.5% vs. 177.9%), and WIS (*p* < 0.001, high vs. low: 6.2%/s vs. 4.3%/s) were significantly larger than those for the low Ki-67 group. No significant difference was observed based on the Ki-67 status for TTP (*p* = 0.183, high vs. low: 40.3 s vs. 45.3 s). For the HR negative group, the MS (*p* = 0.033, negative vs. positive: 20.2%/s vs. 15.9%/s) and WIS (*p* = 0.042, negative vs. positive: 6.1%/s vs. 5.3%/s) were significantly larger than those for the HR positive group. No significant difference was observed based on the HR status for TTE (*p* = 0.334, negative vs. positive: 5.0 s vs. 5.0 s), TTP (*p* = 0.343, negative vs. positive: 40.3 s vs. 45.3 s), and PE (*p* = 0.118, negative vs. positive: 239.1% vs. 202.3%). No significant difference was observed in five UF-MRI kinetic parameters based on the HER2 status (TTE: *p* = 0.496, MS: *p* = 0.818, TTP: *p* = 0.524, PE: *p* = 0.857, WIS: *p* = 0.912). For TNBC, the MS (*p* = 0.028, TNBC vs. non-TNBC: 20.8%/s vs. 16.0%/s) and WIS (*p* = 0.011, TNBC vs. non-TNBC: 6.6%/s vs. 5.3%/s) were significantly larger than those for non-TNBC. No significant difference was noted between TNBC and non-TNBC for TTE, TTP, and PE (TTE: *p* = 0.611, TTP: *p* = 0.064, PE: *p* = 0.084).

**Table 4:** Differences in UF-MRI kinetic parameters according to invasiveness and immunohistopathological characteristics of breast carcinoma.

	TTE (s)	MS (%/s)	TTP (s)	PE (%)	WIS (%/s)
**Invasiveness**					
Invasive (*n* = 62)	5.0 (5.0, 5.1)	17.0 (12.2, 22.2)	42.8 (34.2, 45.3)	221.8 (170.0, 273.5)	5.7 (3.9, 7.3)
Non-invasive (*n* = 16)	7.6 (5.0, 10.1)	9.2 (8.4, 20.3)	45.3 (40.3, 50.3)	154.9 (115.5, 247.4)	3.4 (2.9, 4.7)
p value	*0.029*	*0.040*	0.201	*0.032*	*0.009*
**Ki-67 status**					
high (≥ 14%, *n* = 34)	5.0 (5.0, 5.1)	19.1 (15.9, 23.2)	40.3 (30.2, 45.3)	241.5 (201.7, 302.4)	6.2 (5.1, 8.1)
Low (< 14%, *n* = 28)	5.1 (5.0, 10.0)	12.4 (9.8, 20.6)	45.3 (40.2, 50.3)	177.9 (138.4, 232.2)	4.3 (3.1, 5.7)
p value	*0.020*	*0.004*	0.183	*0.003*	*< 0.001*
**HR status**					
negative (*n* = 15)	5.0 (5.0, 5.1)	20.2 (16.5, 25.3)	40.3 (22.7, 47.8)	239.1 (204.3, 300.2)	6.1 (5.5, 8.4)
positive (*n* = 47)	5.0 (5.0, 10.0)	15.9 (11.5, 21.1)	45.3 (35.2, 45.3)	202.3 (168.5, 264.8)	5.3 (3.8, 6.7)
p value	0.334	*0.033*	0.343	0.118	*0.042*
**HER2 status**					
positive (*n* = 9)	5.0 (5.0, 5.1)	17.6 (10.9, 22.0)	40.3 (40.3, 50.3)	238.0 (170.0, 297.9)	5.9 (5.6, 6.0)
negative (*n* = 53)	5.0 (5.0, 10.0)	16.5 (12.3, 22.2)	45.3 (34.2, 45.3)	219.8 (171.9, 266.1)	5.3 (3.9, 7.3)
p value	0.496	0.818	0.524	0.857	0.912
**TNBC or non-TNBC**					
TNBC (*n* = 12)	5.0 (5.0, 5.1)	20.8 (16.5, 25.3)	37.2 (20.1, 45.3)	247.5 (204.3, 316.6)	6.6 (5.5, 13.2)
Non-TNBC (*n* = 50)	5.0 (5.0, 10.0)	16.0 (10.9, 21.4)	45.3 (35.2, 50.3)	204.3 (168.0, 266.0)	5.3 (3.7, 6.7)
p value	0.611	*0.028*	0.064	0.084	*0.011*

Data are expressed as the median (interquartile range). P-values < 0.05 were considered statistically significant and are underlined. UF-MRI: ultrafast dynamic contrast-enhanced MRI; TTE: time to enhancement; MS: maximum slope; TTP: time to peak; PE: peak enhancement; WIS: wash-in slope; HR: hormone receptor; HER2: Human Epidermal growth factor Receptor 2; TNBC: triple-negative breast cancer.

## Discussion

Our results indicate that the five UF-MRI kinetic parameters for mass and MS, PE, and WIS for NME showed significant differences between malignant and benign breast lesions. The AUCs of the five UF-MRI kinetic parameters for masses (0.69–0.81) and MS, PE, and WIS for NME (0.91–0.94) were comparable to those of BI-RADS categorizations (mass: 0.85, NME: 0.83). The MS and TTE are the two most frequently used kinetic parameters for evaluating the diagnostic performance of UF-MRI^5,6,11,22^. Our results suggest that UF-MRI kinetic parameters other than MS and TTE (including TTP, PE, and WIS) are also useful for distinguishing benign from malignant breast lesions based on both masses and NMEs. The utility of TTP, PE, and WIS in UF-MRI and their diagnostic performances have been reported previously. Cao et al. analyzed 150 BI-RADS categories with ≥ 4 lesions detected in 142 patients using mammography and/or US and reported that TTP was significantly smaller for the malignant lesions (mean, 30.30 s) than for the benign lesions (mean, 54.81 s) (*p* < 0.001). ROC curve analysis revealed that TTP had the highest AUC (0.826; 95% CI: 0.755–0.883) for differentiating benign from malignant lesions^8^. Peter et al. reported the results of a retrospective study involving 61 patients with 83 breast lesions (60 malignant and 23 benign). They reported that PE for UF-MRI was significantly larger for the malignant than for the benign lesions (*p* < 0.0001), and an AUC for PE in differentiating the benign from malignant lesions was 0.781 (95% CI: 0.647–0.897). The sensitivity, specificity, PPV, NPV, and accuracy of PE were 85.0%, 60.9%, 85.0%, 60.9%, and 78.3%, respectively^23^. Cao et al. reported that the AUC of WIS was the highest among those of the five semi-quantitative kinetic parameters in UF-MRI (AUC: WIS, 0.93; TTP, 0.64; bolus arrival time, 0.66; PE, 0.79; and initial AUC in 60 s, 0.91), and the diagnostic performance of WIS was comparable to that of quantitative rate constant, which is the use of contrast agent escape from the extracellular extravascular space into the plasma compartment (0.92) in differentiating malignant from benign lesions (*p* = 0.35)^24^. However, there are few reports on the diagnostic performance of UF-MRI kinetic parameters other than MS and TTE in differentiating malignant from benign breast lesions. In particular, there are very few studies that compare the diagnostic performance of BI-RADS with UF-MRI kinetic parameters and, as in our study, report the diagnostic performance of masses and NMEs.

For mass lesions, BI-RADS demonstrated 100% sensitivity, highlighting its usefulness for detecting breast cancer. However, the sensitivities of UF-MRI kinetic parameters using their optimal cut-off values were significantly lower and their specificities were significantly higher than those of BI-RADS, while their accuracies did not differ significantly. The UF-MRI kinetic parameters tended to yield fewer true positives and false positives and more false negatives and true negatives than BI-RADS for masses (Table 2).

Among the 54 enrolled breast malignant mass lesions, 41 (75.9%) were IDCs. Of these 41 IDCs, the number of true positive lesions by the optimal cut-off values of the five UF-MRI kinetic parameters was 33–38, and the number of false negative lesions was 3–8 (Table 3). Of the IDCs that were false negatives using the optimal cut-off values of the five UF-MRI kinetic parameters (TTE: 7; MS: 3; TTP: 8; PE: 5; WIS: 4) (Table 3), more than one-third of these lesions had irregular shape or margin, or spiculated margins, which are BI-RADS descriptors suggesting malignancy (TTE 7/7; MS 1/3; TTP 7/8; PE 3/5; and WIS 2/4). The morphological characteristics, including the shapes and margins of breast lesions, can help prevent misdiagnosis. Our results suggested that the UF-MRI kinetic parameters cannot replace the overall BI-RADS categorization.

In our study, malignant breast mass lesions included three DCISs. The number of true positives for DCIS using the optimal cut-off values of TTP (3), PE (2), and WIS (3) tended to be higher than that of MS (0) and TTE (1), which were most frequently used for evaluating diagnostic performance of UF-MRI kinetic parameters (Table 3). The TTP, PE, and WIS may be useful for diagnosing DCIS. However, only three DCIS mass lesions were enrolled, and further investigation into more lesions is needed to clarify the details.

Of the benign mass lesions, fibroadenoma tended to yield a higher number of true negatives for UF-MRI kinetic parameters (TTE: 6; MS: 7; TTP: 9; PE: 6; and WIS: 6) than BI-RADS (2) (Table 3). Of the 10 fibroadenomas, 8 lesions were false positives according to BI-RADS categorization. These eight lesions had a fast pattern of TIC on cDCE-MRI. Of these 8 lesions, 4–7 lesions were true positives using optimal cut-off values of UF-MRI kinetic parameters (TTE: 4; MS: 5; TTP: 7; PE: 4; and WIS: 4). Using UF-MRI, tumor vascularity can be examined in more detail in a much shorter time, with lesions that enhance more rapidly or earlier considered more vascular and malignant^25^. The diagnostic performance of fibroadenoma may be improved by adding UF-MRI parameters to evaluate ultra-early blood flow. In one tubular adenoma, both BI-RADS and UF-MRI kinetic parameters yielded zero true positives. Tubular adenomas had smaller TTE (0 s) and TTP (25.2 s) and larger MS (23.5%/s), PE (263.1%), and WIS (10.4%/s) than the other breast lesions. This may be attributable to the tubular adenomas having closely packed round tubules, minimal stroma, and marked blood flow^26,27^. However, as only one lesion was enrolled in our study, a detailed evaluation was challenging.

In our study, the sensitivity, specificity, and accuracy of BI-RADS for mass were 100%, 33.3%, 83.3%, respectively (Table 2); the specificity was low. Some previous studies have reported that combining BI-RADS and ADC value improved specificity and PPV^28,29^. Kul et al. reported that the combination of BI-RADS with the ADC value for differentiating malignant and benign breast lesions (0.92 × 10^− 3^ mm^2^/s) provided 95.7% sensitivity and 89.2% specificity; the specificity of combination of BI-RADS with ADC value improved by 13.5% (*p* = 0.063) compared with that of BI-RADS alone without a significant decrease in the sensitivity (*p* = 1.000)^28^. The diagnostic performance in our study may improve by combining BI-RADS assessment and the ADC value. However, the ADC value was not evaluated in our study because it was not included in the BI-RADS lexicon (5th edition). Moreover, the main purpose of our study was to compare the diagnostic performance of BI-RADS categorization and UF-MRI kinetic parameters.

For NMEs, the sensitivity, specificity, and accuracy of MS, PE, and WIS using their optimal cut-off values were not significantly different from those of BI-RADS. Moreover, the number of true positive and negative NME lesions were similar between BI-RADS and the UF-MRI kinetic parameters (Tables 2 and 3).

For NMEs, no significant difference was observed on TTE (*p* = 0.145, 5.0 vs. 10.0) and TTP (*p* = 0.889, 45.3 s vs. 45.3 s) between malignant and benign lesions, which may be because of the not very high temporal resolution of UF-MRI (5.0 s/phase) and because the rate of non-invasive carcinoma (12 DCIS: 50%) with angiogenesis milder than invasive cancers was higher in malignant NME lesions than in malignant mass lesions (3 DCIS and 1 LCIS: 7.4%). However, the number of enrolled NME lesions (especially benign lesions) was extremely small, and detailed evaluation was difficult.

In our study, the malignant lesions tended to have a larger mean size than benign lesions for both mass (malignant vs. benign: 18.0 mm vs. 14.8 mm, *p* = 0.222) and NME (malignant vs. benign: 29.2 mm vs. 12.5 mm, *p* = 0.032). Some previous studies regarding the diagnostic performance of UF-MRI kinetic parameters for differentiating malignant and benign breast lesions reported that malignant lesions tended to have a larger mean size than benign lesions, which is consistent with our results^5,6^. However, the effects of this finding on the diagnostic performance of UF-MRI kinetic parameters have not been clearly described. Furthermore, some studies have reported that, in malignant lesions, the size and lesion vascularity were related; larger malignant tumors would yield higher perfusion parameters and initial enhancement on UF-MRI because angiogenesis is an essential process for tumor growth^5,30^. Thus, the lesion size may affect the diagnostic performance of UF-MRI kinetic parameters for differentiating between malignant and benign lesions. However, to prove this hypothesis, further research is needed to examine the correlation between detailed pathological findings regarding tumor angiogenesis and the diagnostic performance of UF-MRI kinetic parameters for differentiating between malignant and benign breast lesions.

The TTE (invasive vs. non-invasive: 5.0 s vs. 7.6 s, *p* = 0.029) was significantly smaller, while MS (invasive vs. non-invasive: 17.0%/s vs. 9.2%/s, *p* = 0.040), PE (invasive vs. non-invasive: 221.8% vs. 154.9%, *p* = 0.032), and WIS (invasive vs. non-invasive: 5.7%/s vs. 3.4%/s, *p* = 0.009) for invasive carcinoma were significantly larger than those of non-invasive carcinoma. Several previous studies have reported larger MS and smaller TTE for invasive carcinomas than for non-invasive carcinomas^7,15,31–33^. These findings are consistent with those of our study. Few studies have evaluated the relationship of TTP, PE, and WIS with the invasiveness of breast cancer. Kim et al. reported that PE (their maximum enhancement, invasive vs. non-invasive: 254.8 ± 103.6% vs. 150.5 ± 66.7%, *p* = 0.001) and WIS (their slope, invasive vs. non-invasive: 8.4 ± 4.3%/s vs. 4.9 ± 2.1%/s, *p* = 0.006) for invasive carcinoma were significantly larger than those of non-invasive carcinoma, while TTP showed no significant difference (*p* = 0.44)^34^. These findings are consistent with those of our study. Angiogenesis occurs in hyperplasia and markedly increases during the transition from non-invasive to invasive carcinoma. Invasive carcinoma is associated with higher microvessel density (MVD) than non-invasive carcinoma^35^. Our results may have been influenced by the differences in MVD between invasive and non-invasive carcinomas.

Immunohistopathological findings of invasive breast carcinoma, including high Ki-67 labeling index, HR negative, and TN, are included in poor prognostic factors. In our study, the invasive breast carcinomas with high Ki-67 had significantly larger MS (high vs. low: 19.1%/s vs. 12.4%/s, *p* = 0.004) and shorter TTE (high vs. low: 5.0 s vs. 5.1 s, *p* = 0.020) than those of invasive breast carcinoma with low Ki-67. The invasive breast carcinomas with HR negative and TN had significantly larger MS (HR negative vs. HR positive: 20.2%/s vs. 15.9%/s; *p* = 0.033, TN vs. non-TN: 20.8%/s vs. 16.0%/s; *p* = 0.028) than those of invasive breast carcinoma with HR positive and non-TN. These results were consistent with those of some previous studies^5,36^. Moreover, our findings suggested that PE (high Ki-67 vs. low Ki-67: 241.5% vs. 177.9%, *p* = 0.003) and WIS (TN vs. non-TN: 6.6%/s vs. 5.3%/s; *p* = 0.011, high Ki-67 vs. low Ki-67: 6.2%/s vs. 4.3%/s; *p* < 0.001, HR negative vs. positive: 6.1%/s vs. 5.3%/s; *p* = 0.042), were also associated with immunohistochemical findings of breast carcinoma, in addition to MS and TTE. These results may reflect a high perfusion state^36^ and early strong enhancement^37–39^ in invasive breast carcinoma with poor prognostic factors, including high Ki-67 labeling index, HR negative, and TN. A few reports have examined the details of the relationship between UF-MRI kinetic parameters other than MS and TTE and poor prognostic factors or subtypes^40,41^. Among the five kinetic parameters in UF-MRI evaluated in our study, TTP and PE have been reported to be useful for predicting TN and HER2-positive breast cancer^40,41^. Our results suggested that WIS is useful to predict breast cancer invasiveness, Ki-67, and HR status. We found no previous reports that demonstrated a significant association between WIS and prognostic factors or subtypes. Cao et al. also evaluated the differences among the five semi-quantitative kinetic parameters of UF-MRI including WIS among four subtypes (luminal A, luminal B, TNBC, and HER2-enriched) and the status of prognostic factors including ER (positive vs. negative), PR (positive vs. negative), HR (positive vs. negative), HER2 (positive vs. negative), and Ki-67 (< 20% vs. ≥ 20%). However, neither of the five semi-quantitative kinetic parameters of UF-MRI revealed differences among four molecular subtypes; significant differences were noted only for TTP between ER-positive and ER-negative groups (*p* = 0.04) and between HR-positive and HR-negative groups (*p* = 0.05)^25^. Differences in results for TNBC, HR and Ki-67 of WIS between our study and those of Cao et al. are thought to be due to differences in the definition of analyzed groups (e.g., for subtypes, Cao et al. reported the comparison of each parameter among four subtypes, while we reported the comparisons of TNBC vs. non-TNBC; for Ki-67, Cao et al. reported comparisons between < 20% and ≥ 20%, while we reported comparisons between < 14% vs. ≥ 14%) and differences in UF-MRI scan parameters including temporal resolution (Cao et al. used 4.5 s/phase, representing the first 135 s post-contrast, while we used 5.0 s/phase, representing the first 75 s post-contrast). The WIS depends on the peak height and time to peak, which reflect the tumor perfusion flow rate and blood volume^42^. However, the advantages of WIS over MS and TTE, which are the two most frequently used UF-MRI kinetic parameters, were unclear.

The UF-MRI parameters evaluated in this study, including MS, TTE, TTP, PE, and WIS, reflect the perfusion state and the quantity and distribution of tumor cells in the breast lesions. The pathophysiological mechanisms reflected by each parameter—including tumoral vascular permeability, tumor perfusion flow rate, and blood volume—may differ slightly^8^. These differences may be attributable to the variations in the results for each parameter.

UF-MRI kinetic parameters are affected by imaging techniques (such as CS and TWIST) and scan parameters (including temporal resolution). Our UF-MRI sequence used the view-sharing technique. View-sharing techniques, including TWIST, are effective for categorizing large-scale enhancement patterns. However, because they sample different spatial frequencies at different temporal resolutions, this can introduce errors in quantitative analysis^25^. The CS technique, relative to the view-sharing technique, provides high spatial resolution and reduces blurring. It also enables the acquisition of image data within shorter scan durations than other techniques^43–45^. The CS technique can evaluate kinetic parameters, including TTE and TTP, more accurately than other techniques and is more suitable for their evaluation^45^. However, the view-sharing technique is highly versatile and can be used with several current scanners. It is also the most commonly used technique for UF-MRI.

Despite the above findings, this study had some limitations. First, this was a single-center retrospective analysis, which may introduce selection bias and limit the generalizability of the results. A prospective multi-center study is needed in the future to strengthen the conclusion. Second, few lesions were enrolled; in particular, the sample size of benign NME (*n* = 5) was quite small. This may affect the statistical power and reliability of the findings related to NMEs. More lesions are needed to mitigate post-hoc cut-point bias in deciding optimal cut-off values of UF-MRI kinetic parameters to differentiate between malignant and benign breast lesions. Third, the optimal cut-off values for UF-MRI parameters were derived from the same dataset. External validation or cross-validation is needed to confirm these thresholds. Fourth, a large proportion of the enrolled breast lesions were malignant. Many enrolled patients underwent breast MRI to assess the extent of breast cancer for preoperative evaluation in our institution. Breast lesions imaged to assess tumor extent frequently show more conspicuous malignant features, which may influence BI-RADS assessment and reduce the observed difference in diagnostic performance between UF-MRI and BI-RADS. However, this is attributed to the inclusion of only those cases with a clinical indication for breast MRI. Fifth, our results suggested that the diagnostic performance of UF-MRI kinetic parameters for mass and NME was comparable to that of BI-RADS categorization. However, the specific methods for utilizing these parameters in the clinical workflow have not been clarified. Combining BI-RADS and UF-MRI kinetic parameters may improve diagnostic performance, but further research is needed to clarify the details.

In conclusion, UF-MRI kinetic parameters are useful for differentiating malignant lesions from benign ones. Their diagnostic performance for mass and NME was comparable to that of BI-RADS categorizations. Moreover, UF-MRI kinetic parameters may correlate with the invasiveness and immunohistopathological findings of invasive breast cancer, which influence pre- and postoperative treatment decisions and predict prognosis. However, further studies are needed to support these findings.

## Supplementary Information

Below is the link to the electronic supplementary material.

Supplementary Material 1

Supplementary Material 2

## Data Availability

The datasets generated and/or analysed during the current study are not publicly available due to ethical and legal restrictions but are available from the corresponding author on reasonable request.
