# Supplementary material for: Diagnostic performance of kinetic parameters of ultrafast breast MRI and their associations with immunohistopathological findings of breast carcinoma
Source: Sci Rep. 2026 Jun 2;16:25155. doi: 10.1038/s41598-026-55998-5 (PMC13470266; doi:10.1038/s41598-026-55998-5)
Supplement: Supplementary file 1 [file 41598_2026_55998_MOESM1_ESM.docx]

**Diagnostic performance of kinetic parameters of ultrafast breast MRI and their associations with immunohistopathological findings of breast carcinoma**

**Supplementary Information**

In our study, two patients each had two lesions (101 lesions in 99 patients) which may have introduced within-patient correlation and potentially violated the assumption of independence in lesion-level analyses. To address this concern, we re-analyzed diagnostic performance of BI-RADS and UF-MRI kinetic parameters for masses and NMEs (the main outcomes of this study) on including only the larger lesions in each two patients (i.e., removing two lesions in total and including 99 lesions in 99 patients for the re-analysis) to evaluate the potential impact of within-patient correlation on the results.

The difference in the original results analyzed for all lesions (101 lesions in 99 patients) and re-analysis results analyzed including only larger lesions in the two patients with multiple lesions (99 lesions in 99 patients) was negligible. The re-analysis results are shown in Supplementary Table S1 and Supplementary Fig. S1.

**Supplementary Table S1:**

**Re-analysis results of diagnostic performance comparison of UF-MRI kinetic parameters versus BI-RADS for masses and NMEs**

|  | TP | FN | FP | TN | Sensitivity (%)  (Difference ref.  BI-RADS) | P-value | Specificity (%)  (Difference ref.  BI-RADS) | P-value | Accuracy (%)  (Difference ref.  BI-RADS) | P-value |
| --- | --- | --- | --- | --- | --- | --- | --- | --- | --- | --- |
| Mass | | | | | | | | | | |
| Global test | | | | |  | 0.019 |  | 0.022 |  | 0.728 |
| McNemar’s test | | | | | | | | | | |
| BI-RADS | 53 | 0 | 12 | 6 | 100 |  | 33.3 |  | 83.1 |  |
| TTE | 44 | 9 | 6 | 12 | 83.0 (-17.0) | 0.003 | 66.7 (33.4) | 0.014 | 78.9 (-4.2) |  |
| MS | 46 | 7 | 5 | 13 | 86.8 (-13.2) | 0.008 | 72.2 (38.9) | 0.008 | 83.1 (0.0) |  |
| TTP | 44 | 9 | 6 | 12 | 83.0 (-17.0) | 0.003 | 66.7 (33.4) | 0.034 | 78.9 (-4.2) |  |
| PE | 47 | 6 | 6 | 12 | 88.7 (-11.3) | 0.014 | 66.7 (33.4) | 0.014 | 83.1 (0.0) |  |
| WIS | 49 | 4 | 6 | 12 | 92.5 (-7.5) | 0.046 | 66.7 (33.4) | 0.014 | 85.9 (2.8) |  |
| NME | | | | | | | | | | |
| Global test | | | | |  | 0.159 |  | 0.494 |  | 0.666 |
| McNemar’s test | | | | | | | | | | |
| BI-RADS | 21 | 2 | 2 | 3 | 91.3 |  | 60 |  | 85.7 |  |
| MS | 19 | 4 | 0 | 5 | 82.6 (-8.7) |  | 100 (40) |  | 85.7 (0.0) |  |
| PE | 21 | 2 | 1 | 4 | 91.3 (0.0) |  | 80 (20) |  | 89.3 (3.6) |  |
| WIS | 22 | 1 | 1 | 4 | 95.7 (4.4) |  | 80 (20) |  | 92.9 (7.2) |  |

P-values < 0.05 were considered statistically significant and are underlined.

BI-RADS: Breast Imaging Reporting and Data System; FN: False Negative; FP: False Positive; MS: maximum slope; NME: non-mass enhancement; NPV: Negative Predictive Value; PE: peak enhancement; PPV: Positive Predictive Value; TN: True Negative; TP: True Positive; TTE: time to enhancement; TTP: time to peak; UF-MRI: ultrafast dynamic contrast-enhanced MRI; WIS: wash-in slope

**Supplementary Fig. S1.**

**Re-analysis results of ROC curves and AUCs for BI-RADS and cut-off values of the UF-MRI kinetic parameters for masses (a) and NMEs (b)**

For masses (a), the ROC analysis revealed that the TTE, MS, TTP, PE, and WIS had AUCs ranging from 0.70 to 0.81. A global comparison of AUCs across the six indicators was statistically significant (p<0.001). However, the DeLong’s test showed that the AUCs of UF-MRI kinetic parameters were comparable to that of BI-RADS (0.85) for detecting masses (TTE vs. BI-RADS: p=0.055; MS vs. BI-RADS: p=0.531; TTP vs. BI-RADS: p=0.260; PE vs. BI-RADS: p=0.206; and WIS vs. BI-RADS: p=0.354).

For NMEs (b), the ROC analysis revealed that the MS, PE, and WIS had AUCs ranging from 0.91 to 0.94, which tended to be slightly higher than that of BI-RADS (0.84). The global comparison of AUCs across BI-RADS, MS, PE, and WIS was not statistically significant (p=0.210).

ROC: receiver operating characteristics; TTE: time to enhancement; MS: maximum slope; TTP: time to peak; PE: peak enhancement; WIS: wash-in slope; AUC: area under the curve; UF: ultrafast; BI-RADS: Breast Imaging Reporting and Data System
